# A gene expression profile for detection of sufficient tumour cells in breast tumour tissue: microarray diagnosis eligibility

**DOI:** 10.1186/1755-8794-2-52

**Published:** 2009-08-12

**Authors:** Paul Roepman, Arenda Schuurman, Leonie JMJ Delahaye, Anke T Witteveen, Arno N Floore, Annuska M Glas

**Affiliations:** 1 Agendia BV, Science Park 406, 1098XH, Amsterdam, the Netherlands

## Abstract

**Background:**

Microarray diagnostics of tumour samples is based on measurement of prognostic and/or predictive gene expression profiles. Typically, diagnostic profiles have been developed using bulk tumour samples with a sufficient amount of tumour cells (usually >50%). Consequentially, a diagnostic results depends on the minimal percentage of tumour cells within a sample. Currently, tumour cell percentage is assessed by conventional histopathological review. However, even for experienced pathologists, such scoring remains subjective and time consuming and can lead to ambiguous results.

**Methods:**

In this study we investigated whether we could use transcriptional activity of a specific set of genes instead of histopathological review to identify samples with sufficient tumour cell content. Genome-wide gene expression measurements were used to develop a transcriptional gene profile that could accurately assess a sample's tumour cell percentage.

**Results:**

Supervised analysis across 165 breast tumour samples resulted in the identification of a set of 13 genes which expression correlated with presence of tumour cells. The developed gene profile showed a high performance (AUC 0.92) for identification of samples that are suitable for microarray diagnostics. Validation on 238 additional breast tumour samples indicated a robust performance for correct classification with an overall accuracy of 91 percent and a kappa score of 0.63 (95%CI 0.47–0.73).

**Conclusion:**

The developed 13-gene profile provides an objective tool for assessment whether a breast cancer sample contains sufficient tumour cells for microarray diagnostics. It will improve the efficiency and throughput for diagnostic gene expression profiling as it no longer requires histopathological analysis for initial tumour percentage scoring. Such profile will also be very use useful for assessment of tumour cell percentage in biopsies where conventional histopathology is difficult, such as fine needle aspirates.

## Background

Microarray diagnostics of tumour specimens is based on gene expression measurement of a specific set of predictive or prognostic genes. Bulk tumour samples that consist of tumour cells admixed with surrounding stromal tissue are commonly used for microarray analysis. Although tumour stroma likely plays an important role in tumour development and metastasis [1-3], gene expression profiles are typically generated using tissue that contains sufficient amount of tumour cells, not stroma. Most prognostic gene profiles were originally identified using samples containing at least 50% tumour cells and are, therefore, likely based on gene expression of the neoplastic tissue in question. However, some profiles, e.g. MammaPrint [4,5] have been shown to be accurate with 30% tumour in the diagnostic specimen (AM Glas, unpublished data).

Currently, tumour cell percentage assessment is assessed using heamatoxilin-eosine (H/E) stained specimen for histopathological review. However, even for experienced pathologists, histopathological tumour scoring remains subjective and time consuming and can lead to inconclusive results [6-8] and variable tumour cell percentage scoring. Moreover, tumour cell scoring can be more difficult for core biopsies and especially fine-needle aspirates that are too small or unsuitable for H/E analysis. A tumour percentage scoring method based on tumour cell mRNA transcription levels would provide an additional method to more reliably determine tumour cell content in a reduced time frame.

Herein we report the development of a molecular profile that can accurately identify breast cancer samples with sufficient tumour cell content for diagnostic purpose. This assessment is based on transcription levels and is able to reduce the time that is needed for a microarray experiment as no initial pathological tumour cell percentage (TCP) scoring will be required before sample processing. Moreover, the identified tumour percentage profile would facilitate microarray diagnostics of small specimens for which tumour sections can not be generated for pathological scoring.

## Methods

Four hundred and three frozen tumour samples or tumour samples preserved in RNALater from breast cancer patients were used. At the time of initial diagnosis, all patients had provided consent in the sense that their tumour samples could be used for investigational purposes. The study was carried out in accordance with the ethical standards of the Helsinki Declaration and was approved by the Medical ethical Board of the participating medical centers and hospitals. All samples were de-identified, analyzed anonymously and were part of research implementation studies for MammaPrint.

**Figure 1:**
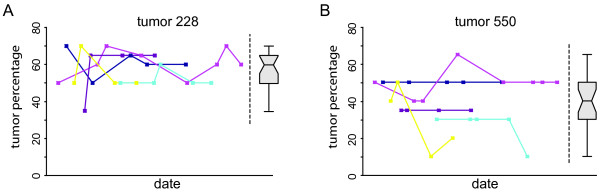
**Variation in histo-pathological tumour percentage scoring**. An identical H/E stained tumour section of two tumours (**A**, T228 and **B**, T550) was scored multiple times by five different pathologists (coloured lines) during a period of 6 months. Box-plots represent the overall pathological variation in TCP scoring for each of the two tumour samples.

Histopathological tumour cell percentage assessment was done based on (H/E) coloured tumour sections. Gene expression analysis of breast tumour samples was performed using custom-made Agilent 44K high-density microarrays according to manufacturer's protocols. Ninety-five selected samples were hybridised against the MammaPrint reference pool (MRP) [5] and included samples with a low (<30%, 27 samples), medium (30–49, 19 samples) and high (≥50%, 49 samples) tumour cell percentage (TCP). Microarray slides were scanned using a Agilent G2565AA scanner and were quantified using Feature Extraction software (version 9.5, Agilent). Gene expression log-ratio data was obtained from non-background subtracted sample/reference signal and was normalised using a lowess global normalisation procedure. A supervised learning approach, similar to that described in [4,9], was applied to design a gene expression profile for the identification of samples with low and high TCP (see below). Potential inter-pathologist variation in tumour cell scoring (+/- 10%, based on Figure 1) was taken into account in the learning model by randomly adjusting the training TCP during each iteration with -10, 0 or 10 percent.

A 3-fold cross validation (CV) method was used to identify a gene expression profile (nearest-mean classifier) that showed a strong association with pathological TCP. Within each CV loop, two-thirds of the samples were randomly selected as training samples and their TCP was adjusted randomly by either -10, 0 or +10 percent to represent pathological variation, as indicated above. For all genes the association between transcriptional levels and continuous pathological TCP was determined (Pearson correlation) and the top 200 genes (100 with positive association and 100 with negative association) were selected for inclusion in the classifier model. This procedure was repeated five-hundred times (multiple sampling approach) and selected genes were ranked according to their CV performance. To identify the optimal number of genes to be used in the classifier model, the top-ranked gene set was gradually expanded and the classifier performance was determined for each gene set size, using leave-one-out CV and measuring the area under ROC curve (AUC). Next, within the set of 35 top-ranked genes, genes were removed that showed a large variation across an additional set of 70 tumour samples with high (≥50%) TCP. Twenty-two genes that showed a variation in gene expression greater than 0.4 (stdev across 70 samples) were excluded from further analysis. This stringent threshold was used to ensure a stable profile in tumours with a relative high tumour cell percentage. The remaining set of 13 genes (Table 1) was used to build a nearest-mean classifier [9]. Optimal threshold setting and performance on all training samples (n = 165) were determined using a leave-one-out CV. The tumour percentage classifier was validated on 238 independent breast tumour samples.

**Table 1:** Genes used for building a tumour cell percentage associated profile.

**Gene**	ID*	**Description**
AI732974	AI732974	Homo sapiens cDNA clone IMAGE:1473008 3'
AK025430	AK025430	Homo sapiens cDNA FLJ21777 fis, clone HEP00173
AK094860	AK094860	Homo sapiens cDNA FLJ37541 fis, clone BRCAN2026340
ANAPC7	NM_016238	Homo sapiens anaphase promoting complex subunit 7
BC031974	BC031974	Homo sapiens cDNA clone IMAGE:4837645
C17ORF73	ENST00000300458	Homo sapiens mRNA for hypothetical protein FLJ20694 variant, clone: COL06209
HEATR3	NM_182922	Homo sapiens HEAT repeat containing 3
LOC147804	NM_001010856	Homo sapiens hypothetical protein LOC147804
OGT	NM_181672	Homo sapiens O-linked N-acetylglucosamine (GlcNAc) transferase
PRR13	NM_001005354	Homo sapiens proline rich 13 (PRR13)
SNAP29	NM_004782	Homo sapiens synaptosomal-associated protein, 29 kDa
CNPY3	AF161347	Homo sapiens HSPC084 mRNA, partial cds
TPM3	NM_152263	Homo sapiens tropomyosin 3 (TPM3)

* ID: Refseq, Genbank or Ensembl identifier.

Biological function analysis was performed using Ingenuity Pathway Analysis (IPA version 6.3, Ingenuity Systems Inc, Redwood City, CA). Full genome gene expression measurement of all samples analysed in this study is publicly available at the Gene Expression Omnibus (GEO) with accession number GSE16201.

## Results

### Pathological tumour cell percentage related gene expression in breast cancer

To determine the variation in histopathological tumour cell percentage scoring, H/E stained tumour sections were repeatably analysed in a period of six months by five different pathologists (Figure 1). Although the variation between pathologists (10–15%) was larger than the variation observed in repeated scoring over time by the same pathologist (5–10%), the average variation in tumour cell percentage scoring was 10 percent (range 0 to 60%). This observed pathological variation (10%) is taken into account for classifier development as described in detail in the materials and methods.

Using 95 breast tumour samples that ranged in tumour cell content from zero to 85 percent, based on analysis of HE slides, a tumour percentage gene expression profile was identified. A 3-fold cross validation (CV) method was used to identify a gene expression profile (nearest-mean classifier) that showed a strong association with pathological TCP. This procedure was repeated five-hundred times (multiple sampling approach) and genes were ranked according to their CV performance. The multiple sampling CV performance indicated a strong association of the selected nearest-mean classifier model with pathological TCP (R2 = 0.42, P < 0.001, Wilcoxon rank-sum test). To identify the optimal/minimal number of genes to be used in the classifier model, the top-ranked gene set was gradually expanded and the classifier performance (area under ROC curve, AUC) was determined for each gene set size.

**Figure 2:**
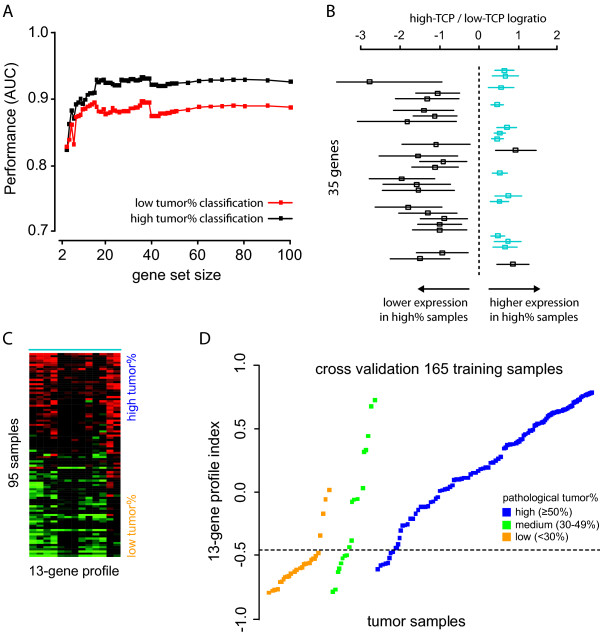
**Identification of a tumour percentage transcriptional profile**. (**A**) Classifier performance (area under ROC curve) using different top-ranked gene set sizes. Performance is given for classification of low (<30%) and high (≥50%) TCP samples. (**B**) Differential gene expression of top 35 ranked genes between low and high TCP samples. Bars represent expression variation of each gene across 70 high TCP samples. Thirteen most stably differential expressed genes for high TCP are indicated in blue. (**C**) Heat-map of the 13-gene tumour percentage profile for 95 training samples. Samples are ordered according to their pathologist scored tumour percentage. (**D**) Profile outcome for all 165 training samples. Profile indexes are calculated using a leave-one-out CV. Tumour samples are grouped according to their tumour percent scoring and ordered according to the profile index. Horizontal dashed line represents the optimal classification threshold.

The classifier showed a strong performance (AUC >0.88) when at least ten of the identified TCP associated genes were included in the classifier model (Figure 2A). The highest performance was reached upon inclusion of the top 35 genes in the classifier and correctly scored 90 percent of the low TCP (<30%) samples and 93 percent of the high TCP (≥50%) samples.

### Identification of a 13-gene profile for assessment of TCP

Although optimal performance was reached with the complete set of 35 genes, a substantial number of genes from the 35-gene set showed a significant single-gene performance (AUC >0.80, 12 genes; AUC >0.80, 15 genes). A minimal combination of just two random genes from within the 35-gene set resulted in an AUC of greater than 0.80. This performance increased to >0.85 for random set of least 5 genes (data not shown).

In a next step to further optimise the classifier, an additional set of 70 tumour samples was used to select from the 35 gene set those genes that showed stable expression in samples with a high tumour percentage. As expected, genes with a higher expression for high TCP (Figure 2B, right-side) showed a more stable expression across the 70 additional high TCP samples (Figure 2B, blue bars). The 13 genes with the most stable expression (Table 1), which were all up-regulated in high versus low TCP (Figure 2B), were selected to build a robust tumour percentage expression profile (Figure 2C). The 13-gene classifier showed a high performance (AUC 0.92) for identification of samples suitable for microarray diagnostics (Table 2). Use of the optimal classifier threshold for maximum sensitivity and specificity resulted in accurate classification of 85 percent of low TCP samples and 93 percent of high TCP samples (Figure 2D).

**Table 2:** Tumour cell percentage profile (TCP) performance.

	**Median TCP profile index**	**P-value**	**Accuracy**
**Training cohorts (n = 165)**			
low pathological TCP (<30%)	-0.592		85%
medium/high pathological TCP (≥30%)	0.327	P < 0.0001	88%
Kappa score: 0.68 (95% 0.50 – 0.72)			

**Validation cohort (n = 238)**			

low pathological TCP (<30%)	-0.589		78%
medium/high pathological TCP (≥30%)	0.305	P < 0.0001	93%
Kappa score: 0.63 (95%CI 0.47–0.73)			

P-value, Student's T-test; TCP Accuracy, accuracy between TCP profile assessment and pathological scoring.

Biological function analysis indicated that the 35 TCP associated gene set was enriched for genes associated with cancer related processes such as cellular morphology (*MGAT3*, *AQP1*, *TPM3*, *MYLK*, *SFRP1*, *NTRK2*: p = 1.7e-3), cell-to-cell signalling (*JAM2*, *LOC338328*, *OGT*, *MYLK*, *NTRK2*, *SNAP29*: P = 1.7e-3), cellular movement and invasion (*MGAT3*, *TPM3*, *MYLK*, *SFRP1*, *NTRK2*, *CCL15*: P = 1.7e-3) and cellular survival and apoptosis (*TPM3*, *PRR13*, *OGT*, *MYLK*, *SFRP1*: P = 3.6e-5).

### Validation of the 13-gene profile on a cohort of 238 breast tumour samples

**Figure 3:**
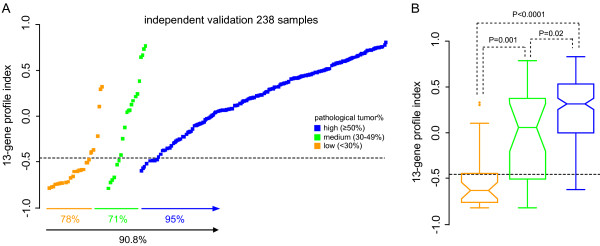
**Validation of the 13-gene tumour percentage profile on 238 independent samples**. (**A**) Profile outcome for 238 independent validation samples. Tumour samples are grouped according to their pathological scoring and ordered according to the profile index. Horizontal line represents the classification threshold that has been determined on the training cohort. (**B**) Box-plots and statistical differences in profiles indexes between the pathological low, medium and high TCP groups. Colours similar as in **A**.

The developed tumour percentage profile was validated on an independent set of 238 breast tumour samples of varying tumour cell percentage (range 0–90%). The TCP distribution across this validation cohort was representative of typical diagnostic samples. The 13-gene molecular TCP profile correctly assigned 78, 71 and 95 percent of the low, medium and high TCP samples as assessed by pathological scoring (Figure 3A). The profile showed an overall accuracy of 91 percent and a kappa score of 0.63 (95%CI 0.47–0.73) and indicating a strong association between both methods (Table 2). The profile showed a false-positive classification rate of 5% (high-TCP samples classified as low by the profile) and a false-negative classification of 21% (low-TCP samples classified as high by the profile).

Analysis of continuous TCP profile index and continuous pathological tumour cell percentage scoring resulted in a significant association between both methods (R^2 ^= 0.48, Wilcoxon P < 0.001, AUC 0.90) and indicated that the profile might also be useful for indication of accurate tumour cell percentage instead of a low-, medium- or high-TCP classification. Statistical analysis of the profile indexes indicated a significant difference in outcome between samples with a low and medium TCP (P = 0.01, Student's T-test) and between samples with a low and high TCP (P < 0.0001) (Figure 3B). Although the difference in index between medium and high TCP samples was borderline significant (P = 0.02), the majority of medium TCP samples (78%) were classified as high TCP by the molecular profile. This result indicates that both medium and high TCP harbour a different tumour cell related gene profile compared to low TCP samples. The validation results confirm that the molecular profile can accurately determine samples with a sufficient tumour percentage for microarray diagnostics based on transcriptional analysis of 13 identified genes.

## Discussion

This study reports the development of a tumour cell percentage (TCP) assessment method for breast cancer samples based on transcriptional analysis of 13 genes. The 13-gene molecular profile has been validated on an independent cohort of 238 samples and can accurately identify breast tumour samples with a sufficient number of tumour cells for microarray diagnostics. Tumour percentage scoring based on the molecular profile is identical to the pathologist scoring for more than 90 percent of all analysed tumour samples. Although the variation in pathologist TCP scoring is in agreement with the number of discrepancies between pathological and molecular profile classification, this inconsistency is likely caused by the difference between the number of tumour cells present in a tumour sample and the actual tumour cell specific mRNA levels. In addition to the known variation and inconsistency in pathological scoring of tumour slides (e.g. Figure 1 and [6,7]), Hsu *et al. *indicated that formalin fixation of slides can result in tumour cell shrinkage [10] and can lead to an underestimation of tumour cell content. Since the TCP profile is based on transcriptional levels of high TCP related gene expression within fresh or frozen tumour tissue, we believe that the developed gene profile likely gives a better indication whether a sample is suitable for microarray diagnostics compared to a pathological tumour cell percentage scoring on formalin-fixed H/E stained slides.

The utility of the gene profile lies in its capability to identify tumours with a high percentage of tumour cells compared to tumours with insufficient tumour content for subsequent microarray diagnostics. Conventional histopathological review results in tumour cell percentage scorings up to 10% increments but is laborious and requires an experienced (in-house) pathologist. The profile, on the other hand, is able to distinguish samples with low-, medium- or high-tumour cell content. Although the 13-gene profile provides a more qualitative measurement compared to the quantitative pathological assessment, it is a value tool for an objective TCP scoring based on transcriptional levels that can quickly identify samples suitable for diagnostics.

Since the gene profile was developed in such a way to mimic a pathological scoring which has been described as inconsistent and subjective, one might argue that the TCP profile also suffers from these factors. The main goal of this study, however, was not to develop a more accurate tool for TCP assessment but an objective method that can be performed independent of pathological expertise and which is based on transcriptional gene levels instead of the number of tumour cells. The use of a robust cross validation procedure that included a mimicked pathological variation was therefore included in the selection model that should, in principle, select a set of genes that are robust to this variation. Nevertheless, the 10 percent misclassification between the gene profile and pathological scoring might partly be attributed to this phenomenon.

Future application of the developed TCP profile could significantly improve the throughput of microarray diagnostics. Currently, after sample arrival, RNA processing and expression analysis cannot proceed until a histopathological TCP analysis confirms that the tumour sample contains sufficient tumour cells for analysis. As indicated above, pathological analysis will remain necessary for detection of ductal carcinoma in situ, necrosis and a detailed assessment about the percentage of tumour cells to define the suitability of the specimen. However, replacement of initial pathologist TCP scoring by a faster transcription based analysis that is able to identify whether a sample likely contains sufficient tumour tissue will shorten the processing time for microarray diagnostics. More importantly, the 13-gene molecular profile for tumour cell percentage (TCP) enables gene expression diagnostics for small samples or those on which no H/E evaluation is possible.

While gene expression profiles tend to be more robust with inclusion of a larger set of genes [9], we decided to limit the developed profile to a relatively small number of genes with optimal performance. The rationale behind this strategy was that transcriptional TCP assessment is preferably done before diagnostic microarray analysis as this assessment will indicate whether a sample is qualified for gene expression profiling. Future development of the 13-gene molecular profile into a RT-qPCR based assay, will allow a less subjective qualification of breast tumour samples as suitable for microarray diagnostics. This way, only samples with a sufficient TCP will be used for microarray diagnostics, saving time, money, and eliminating the need for a pathologist to score TCP on qualified specimens.

## Conclusion

Whether one uses a qPCR assay for specimen selection or uses parallel microarray readout for final approval, the developed 13-gene TCP profile will provide an additional tool for objective assessment of sufficient tumour cell content in breast cancer tissue. It will improve the efficiency and throughput for diagnostic gene expression profiling as it eliminates the need for histopathological analysis in initial tumour percentage scoring. Moreover, it will also allows likely qualify small clinical samples, such as fine-needle aspirates, for transcriptional diagnostics.

## Competing interests

All authors are employed by Agendia BV. PR and AMG have filed a patent application for the use of the tumour cell percentage profile.

## Authors' contributions

PR, ANF and AMG were involved in development of the study design. AS, LJMJD and ATW performed the microarray gene expression analysis. PR performed the data analysis and PR, ANF and AMG were involved in interpretation of the results. PR and AMG participated in writing the manuscript and all authors have read and approved the final manuscript.

## Pre-publication history

The pre-publication history for this paper can be accessed here:


